# Paroxysmal Atrial Fibrillation Triggered By A Monomorphic Ventricular Couplet In A Patient With Acute Coronary Syndrome

**DOI:** 10.1016/s0972-6292(16)30460-0

**Published:** 2012-01-31

**Authors:** Luca De Mattia, Marco Brieda, Ermanno Dametto, Federica Del Bianco, Gian Luigi Nicolosi

**Affiliations:** Department of Cardiology ARC, Azienda Ospedali Riuniti del Pordenonese, Pordenone, Italy

**Keywords:** Atrial fibrillation, acute coronary syndrome, ventricular premature beat, myocardial ischemia, adrenergic system

## Abstract

Atrial fibrillation is a common arrhythmia in patients suffering from acute myocardial infarction, however its pathophysiological mechanisms are not fully understood. We describe the unusual case of a 76-year old woman admitted for non-ST-segment elevation myocardial infarction, who developed multiple episodes of paroxysmal atrial fibrillation triggered by monomorphic ventricular couplets. Beta-blocking and amiodarone therapy resulted efficacious in preventing arrhythmic recurrences. We then discuss the possible arrhythmogenic mechanisms, with special emphasis on the unique electrophysiological, hemodynamic, cellular and anatomical milieu created by acute myocardial ischemia.

## Introduction

Atrial fibrillation (AF) is a common arrhythmia in patients with acute coronary syndrome and carries a negative prognostic value [1]. The pathophysiological mechanisms are complex and not fully understood, yet there is overwhelming evidence that AF episodes are commonly triggered by ectopic atrial premature beats.

We report the case of a 76-year old woman with a non-ST-segment elevation myocardial infarction (NSTEMI) who developed multiple episodes of sustained AF triggered by premature monomorphic ventricular couplets.

## Case Report

A 76-year old obese woman presented to the intensive care unit for acute NSTEMI. Her medical history included poorly-controlled insulin-dependent diabetes and systemic hypertension. Two hours before the admission, pain in the substernal area developed suddenly. The first electrocardiogram obtained showed normal sinus rhythm with a rate of 85 beats per minute and ST-segment depression of 3 mm in the inferior leads. Serum levels of hematocrit, creatinine and electrolytes were all within the normal range. The troponin I level was 6.4 ng per milliliter (normal range < 0.05 ng per milliliter). Nitroglycerin, morphine, loop diuretics, unfractioned heparin and tirofiban IV were administered and the patient became asymptomatic shortly thereafter. Oral therapy with clopidogrel 75 mg, simvastatin 20 mg and metoprolol 50 mg bid was added.

Trans-thoracic echocardiography showed a normal left ventricular ejection fraction of 55% in the absence of segmental wall motion abnormalities, mild hypertrophy of the left ventricle, mild left atrial enlargement (transverse diameter 4.4 cm) and moderate mitral regurgitation.

**Figure 1:**
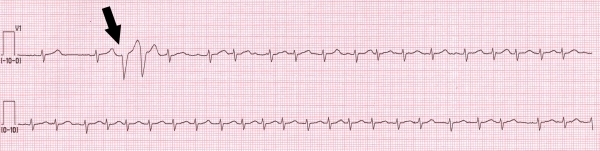
ECG monitoring tracing recorded during the acute phase of NSTEMI (V1 precordial lead). During sinus rhythm two ventricular premature beats with a coupling interval of 280 msec (arrow) elicit onset of atrial fibrillation with irregular ventricular response.

**Figure 2:**
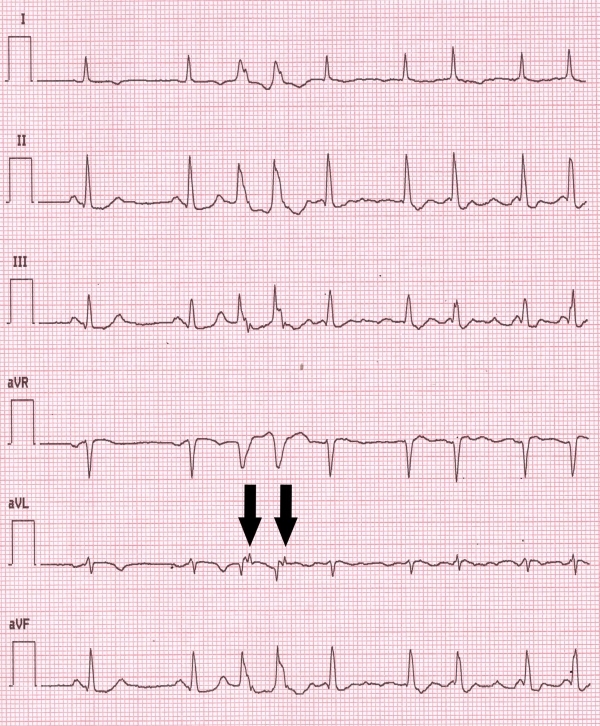
The same AF episode as in Figure 1, limb leads. Note the presence of a positive P wave in aVL lead and a negative P wave in D3 lead following each ventricular premature beat (arrows), suggesting a retrograde atrial activation through the AV node.

Continuous ECG monitoring showed multiple episodes of sustained, self-terminating paroxysmal AF (Figure 1) symptomatic for dizziness, hypotension and palpitations. All AF episodes were triggered by short-coupled (280 msec coupling interval) ventricular monomorphic couplets with 1:1 ventriculo-atrial (VA) retrograde conduction. The ventricular ectopic beats showed left bundle-branch block (LBBB) morphology with inferior axis (Figure 2, arrows) and retro-conducted P waves were detectable in aVL and inferior leads (Figure 2, arrows). Intravenous metoprolol (5 mg) and amiodarone (900 mg) were administered and in one hour stable sinus rhythm was achieved.

The day after a coronary angiography was performed, which showed severe right coronary ostial stenosis. Balloon angioplasty of the lesion was performed and a bare-metal stent was successfully deployed.

The patient was discharged home four days later in therapy with acetyl-salicylic acid 100 mg, clopidogrel 75 mg, furosemide 25 mg, metoprolol 50 mg bid, enalapril 20 mg, atorvastatin 80 mg and insulin. A pre-discharge echocardiography was unchanged. At 1 month follow-up visit she was in stable NYHA functional class II, asymptomatic for angina and palpitations and in sinus rhythm.

## Discussion

Atrial fibrillation is the most frequently encountered arrhythmia in clinical practice, both in the outpatient population and in patients with acute coronary syndrome [2]. The incidence of AF among acute MI patients varies between 2% and 22%, being more frequent in older patients and in those with left ventricular dysfunction. AF in the setting of acute MI is associated with increased in-hospital and 1-year mortality and risk of stroke [1].

There is overwhelming evidence that AF in the general population is commonly triggered by atrial ectopic beats arising from foci inside the pulmonary veins or, less frequently, other atrial structures, [3] in the presence of a favourable substrate (mainly atrial fibrosis and loss of atrial muscle mass) [2]. In other settings (e.g. in acute coronary syndromes or in patients with Wolff-Parkinson-White syndrome (WPW) due to the presence of an accessory atrio-ventricular (AV) pathway) the pathophysiological mechanisms may be different.

In the setting of acute coronary syndromes the substrates for the development of AF are electrophysiological (dispersion of atrial refractoriness), cellular (acute channel expression change) and anatomical (atrial tissue oedema and ischemia/necrosis, atrial stretch), whereas triggers have not been extensively studied, although atrial premature beats can intuitively play a major role [1].

To the best of our knowledge this is the first description of a patient who developed AF episodes triggered by monomorphic ventricular couplets in the acute phase of NSTEMI. Interestingly, Agarwal et al recently reported that in a community study examining a large cohort of non-ischemic, middle-aged patients, frequent premature ventricular beats were associated with an increased risk of non carotid embolic stroke: according to these data one may speculate that, at least in some of these patients, ventricular arrhythmias could have triggered AF episodes which in turn lead to embolic stroke [4].

Peinado and colleagues previously reported the case of a patient with WPW and chronic ischemic heart disease who developed AF triggered by ventricular couplets during an electrophysiological study for ablation of the accessory pathway [5]. The authors recorded intracardiac electrograms showing AF episodes initiated by monomorphic ventricular couplets with retrograde ventriculo-atrial (VA) conduction through a left posterior accessory pathway. Shen et al also described the case of a patient with a concealed form of Wolff-Parkinson-White syndrome where AF episodes were triggered by isolated ventricular premature beats with a short coupling interval, whereas ventricular premature beats with the same morphology but longer coupling interval initiated AV reciprocating tachycardia [6]. In patients with WPW the presence of an AV accessory pathway can per se create the anatomic substrate for AF onset [7]. Moreover, the unique electrophysiological properties of AV accessory pathways (ie absence of decremental conduction properties and short effective refractory period) allow the electrical activation originating from ventricular premature beats to be rapidly conducted to the atria during the vulnerable period, thus triggering AF.

Although intracardiac electrophysiological study was not performed (we considered unethical to perform it in our patient, since it would not have added any prognostic or therapeutic information), the presence of an accessory AV pathway did not seem likely, given the absence of pre-excitation at baseline ECG, history of palpitations episodes or documented reciprocating tachycardia episodes. Even though intracardiac electrograms were not obtained, the short VA conduction time (80 msec), the positive polarity of retro-conducted P waves in aVL lead and their negative polarity in D3 lead (Figure 2, arrows), were again highly suggestive of retro-conduction through the AV node [8].

The morphology of the ventricular ectopic beats recorded from limb leads (LBBB with inferior axis; Figure 1, arrow) suggested a right ventricular outflow tract (RVOT) origin. Nuria Rivas et al demonstrated that during isoproterenol infusion (a condition simulating the high adrenergic tone which occurs in acute MI patients) the most common site of origin of premature ventricular beats is RVOT [9]. Another possible mechanism to explain the RVOT origin of ventricular ectopic beats in this patient is the presence of myocardial ischemia due to severe right coronary ostial lesion, which usually gives off the conus arteriosus artery to supply blood to the RVOT. The right coronary artery (together with the circumflex artery) usually gives off branches to the atria, thus creating another possible ischemic substrate for AF development in our patient.

The enhanced sympathetic tone may also have shortened AV node refractory period, nodal conduction time and attenuated decremental conduction properties of AV node, which usually protects the atria during the electrical vulnerable period. Moreover, premature atrial contractions against closed AV valves may have increased atrial stretch, thus creating another favourable condition for the development of atrial arrhythmias. Metoprolol and amiodarone (which share antiarrhythmic, beta-blocking and anti-ischemic properties) may have played a synergistic effect in blocking ventricular ectopies and AF episodes.

## Conclusions

AF is a common arrhythmia in the setting of acute coronary syndrome and carries important prognostic significance; nevertheless its pathophysiological mechanisms in this particular setting are not thoroughly understood.

We describe the unusual case of a patient who developed AF episodes triggered by ventricular couplets with likely nodal retrograde VA conduction in the absence of overt accessory AV pathways. The enhanced sympathetic tone which characterizes the acute phase of MI and the presence of myocardial ischemia may have played a major role both in creating a favourable substrate and triggering AF episodes. In this setting beta-blockers and amiodarone may have acted with a synergistic anti-adrenergic, anti-ischemic and antiarrhythmic effect.
